# Revolutionizing Orthopedic Healthcare: The Role of Robotics

**DOI:** 10.7759/cureus.44820

**Published:** 2023-09-07

**Authors:** Pothuri R Ram, Madhan Jeyaraman, Naveen Jeyaraman, Sankalp Yadav, Ravichandran Venkatasalam

**Affiliations:** 1 Orthopaedics and Trauma, Sanjay Gandhi Institute of Trauma and Orthopaedics, Bengaluru, IND; 2 Orthopaedics, ACS Medical College and Hospital, Dr. MGR Educational and Research Institute, Chennai, IND; 3 Medicine, Shri Madan Lal Khurana Chest Clinic, New Delhi, IND; 4 Regenerative Medicine, Orange Healthcare, Chennai, IND

**Keywords:** ethical considerations, medical innovation, surgeon-robot collaboration, musculoskeletal conditions, robotics, ai and robotics in healthcare

## Abstract

Integrating robotics into orthopedic healthcare represents a transformative paradigm shift driven by technological advancements. This editorial explores the profound impact of robotics on the diagnosis, treatment, and rehabilitation of musculoskeletal conditions. Robotics redefines precision in orthopedic surgery through advanced imaging and real-time feedback, resulting in minimized disruption to tissues and faster recovery. Personalized treatment plans leverage robotics' capabilities to tailor procedures to individual anatomical characteristics, enhancing outcomes and reducing complications. Minimally invasive procedures, facilitated by robotics, mitigate trauma and expedite patient recovery. This collaboration between surgeons and robotic systems enhances precision without supplanting human expertise. Moreover, robotics extends to postoperative rehabilitation, utilizing exoskeletons and motion-capture systems to optimize mobility and strength recovery. While challenges of cost and training exist, proactive collaborations are shaping the future of robotics in orthopedic care. Ethical considerations underline the importance of balancing human intervention with robotic assistance. As robotics evolves, orthopedic healthcare embraces a future where technology and human expertise synergize, ultimately conquering musculoskeletal conditions.

## Editorial

In recent years, the realm of healthcare has been undergoing a transformative journey fueled by remarkable technological advancements. Among the most impactful areas experiencing this revolution is orthopedic healthcare, where robotics catalyzes a paradigm shift in approaching the diagnosis, treatment, and rehabilitation of musculoskeletal conditions. As the symbiotic relationship between robotics and orthopedic care continues to evolve, a future brimming with the potential for enhanced precision, swifter recovery times, and overall improved outcomes comes into view.

Orthopedic conditions, an extensive array of musculoskeletal disorders, have long posed challenges due to the intricate complexity of human anatomy and the delicate nature of bone and joint structures. Robotics' assimilation into orthopedic care has introduced a new dimension, addressing these challenges with unprecedented precision and efficacy.

Robotic systems revolutionizing orthopedics

Several robotic systems have emerged as game-changers in orthopedic surgery, each offering unique features and benefits.

MAKO Surgical System

The MAKO system (Stryker Corporation, Kalamazoo, Michigan) is renowned for its application in joint replacement surgeries. Advanced imaging and real-time feedback assist surgeons in precise bone preparation and implant positioning, resulting in improved alignment and joint function. Its haptic feedback mechanism aids in accurate bone resection. MAKO utilizes computed tomography (CT) scans to create patient-specific 3D models. Surgeons plan implant placement virtually, ensuring optimal alignment. The robotic arm is guided by the surgical plan in the operating room and provides haptic feedback to prevent excessive bone resection. Surgeons retain control while benefiting from enhanced accuracy.

ROSA (Robotic Surgical Assistant)

ROSA (Zimmer Biomet, Warsaw, Indiana) is widely utilized in neurosurgery and spine procedures. Its features include real-time intraoperative data, image guidance, and robotic arms for enhanced surgical precision. ROSA facilitates minimally invasive surgeries and assists in complex spinal deformity corrections. ROSA integrates with various imaging modalities like CT and X-ray, to create a comprehensive surgical plan. It assists in cranial and spinal surgeries by providing real-time data, enabling precise targeting and trajectory planning. The system's robotic arms are guided by the surgeon's commands, enhancing procedural accuracy.

NAVIO Surgical System

NAVIO (Smith & Nephew, Watford, England) offers robotics-assisted procedures in orthopedics, focusing on knee surgeries. It employs intraoperative planning and a handheld robotic-assisted tool for precise bone resurfacing. NAVIO's image-free registration enhances accuracy and outcomes in knee arthroplasty. NAVIO operates without preoperative imaging, utilizing a computer-assisted tool for intraoperative planning. Surgeons use tactile guidance to create a patient-specific plan and execute bone resurfacing accurately. This system enhances implant alignment, potentially improving implant longevity and patient satisfaction.

Stryker Mako Total Hip Arthroplasty System

The Stryker Mako total hip arthroplasty system (Stryker) is tailored specifically for total hip arthroplasty. It provides a combination of 3D preoperative planning and robotic arm assistance during surgery. Surgeons benefit from real-time feedback, facilitating accurate acetabular cup positioning and leg length restoration. This system combines preoperative planning with intraoperative robotic assistance. Surgeons use a handheld robotic arm to prepare the acetabulum and place the cup precisely. Real-time feedback ensures accurate leg length restoration and hip offset, minimizing complications.

RAS (Robotic Arm System) in Spine Surgery

RAS systems (RAS Systems, LLC, Peachtree City, Georgia) are designed to enhance the accuracy of pedicle screw placement in spine surgeries. They utilize advanced imaging and robotic arms to guide surgeons in complex procedures, reducing the risk of nerve injury and revision surgeries. RAS employs advanced imaging and navigation to guide robotic arms for pedicle screw placement. Surgeons create a preoperative plan, and the robotic arm assists in accurate trajectory execution. This reduces radiation exposure, enhances accuracy, and potentially reduces revision surgeries.

MAZOR X Stealth Edition^TM ^Robotic Guidance System

The MAZOR X Stealth™ Edition robotic guidance system (Medtronic plc, Minneapolis, Minnesota) offers both preoperative and intraoperative planning capabilities. With features like customizable implant options, optimal trajectories for implants, and advanced 3D analytics, this planning functionality empowers surgeons to enhance construct optimization and achieve a more predictable surgical procedure. The foundation of robotic precision lies in cutting-edge registration and mechanical stability. The MAZOR X™ platform establishes a closed-loop connection between the robotic arm mounted on the operating table, the securely positioned patient, and a rigid link between the robot and the patient's skeletal structure. This robotic system is table-mounted, optimizing the operating room space while ensuring unparalleled precision. The Stealth™ Navigation technology provides real-time visualization of implant placement within the patient's anatomy, aligning with the pre-operative plan. By seamlessly integrating two guidance technologies into a comprehensive platform, surgeons can confidently execute procedures during surgery, effectively bridging the gap between planning and execution. The navigation feature offers the visibility required to complete the surgical plan. With an accuracy rate of up to 100%, the MAZOR X Stealth™ Edition ensures precise screw placement.

Precision redefined

Among the most compelling contributions of robotics to orthopedics is its capacity to elevate surgical precision. Conventional surgical techniques often hinge on the surgeon's manual dexterity, leaving room for inadvertent errors. Robotic systems, fortified with advanced imaging and real-time feedback mechanisms, empower surgeons to meticulously plan and execute procedures with a degree of accuracy measured in sub-millimeters [1]. This level of precision translates into minimal disruption to surrounding tissues, resulting in diminished post-operative discomfort and expedited patient recovery periods following surgeries like spine surgeries and arthroplasty.

Personalized treatment plans

Introducing robotics in orthopedic surgery marks a revolutionary phase in tailored treatment plans. Robotic systems enable surgeons to intricately strategize procedures that cater to each patient's unique anatomical characteristics [2]. This high level of personalization significantly improves surgical outcomes and diminishes the likelihood of complications, consequently minimizing the need for subsequent corrective interventions.

Minimally invasive procedures

Robotics has been instrumental in fostering the proliferation of minimally invasive procedures within orthopedics [3]. Conventional open surgeries often entail sizeable incisions and protracted recovery periods. Robotic-assisted surgeries, on the other hand, necessitate smaller incisions, thereby reducing trauma to the surrounding tissues [4]. This results in diminished pain, abbreviated hospital stays, and an expedited return to everyday activities. Patients are no longer required to endure prolonged recovery periods, as robotics swiftly empowers them to reintegrate into everyday routines.

Advantages of robotic systems in orthopedics

The following are the advantages of robotic systems in orthopedics.

Enhanced precision: Robotic systems offer unmatched precision and accuracy in executing surgical tasks, leading to improved implant alignment and surgical outcomes.

Personalized treatment: Robotic platforms allow for the customization of surgical procedures based on each patient's unique anatomy, potentially reducing complications and improving long-term outcomes.

Minimized tissue trauma: Robotic-assisted procedures often require smaller incisions and result in less disruption to surrounding tissues, leading to reduced postoperative pain and faster recovery times.

Improved implant placement: Robotic systems enable precise implant positioning, potentially increasing implant longevity and patient satisfaction.

Real-time feedback: Many robotic platforms provide real-time feedback to surgeons during procedures, allowing for adjustments and ensuring optimal outcomes.

Complex maneuvers: Robotic arms can execute complex maneuvers that might be challenging for a human hand, enhancing the surgeon's capabilities.

Reduced radiation exposure: In procedures such as spine surgery, robotics can reduce the need for fluoroscopy, leading to lower radiation exposure for both patients and surgical staff.

Disadvantages of robotic systems in orthopedics

The following are the disadvantages of robotic systems in orthopedics.

High initial costs: Robotic systems require substantial investments in terms of equipment and training, potentially increasing the overall cost of procedures.

Learning curve: Surgeons need to undergo specialized training to operate robotic systems effectively, and the learning curve can initially extend procedure times.

Limited flexibility: Robotic systems follow preplanned trajectories, which might limit the surgeon's ability to make spontaneous adjustments during the procedure.

Dependency on technology: Technical malfunctions or software glitches could interrupt surgeries or lead to suboptimal outcomes, highlighting the system's reliance on technology.

Time-consuming setup: Setting up the robotic system and registering patient data can add time to the overall surgical process, especially during the learning phase.

Complexity of integration: Integrating robotic systems into existing surgical workflows might be challenging and require adjustments in hospital processes.

Ongoing maintenance and costs: Beyond the initial investment, robotic systems require maintenance, software updates, and potential hardware upgrades, leading to ongoing costs.

Surgeon-support collaboration

A fundamental tenet of understanding robotics in orthopedics is that it augments, rather than supplants, the role of surgeons. Robotic systems are invaluable tools, furnishing real-time insights, haptic feedback, and unparalleled visualization during procedures. Surgeons retain complete control while benefiting from the precision and data-driven guidance offered by the robotic platform [5]. This collaborative alliance between human expertise and robotic precision synergistically pushes the boundaries of what orthopedic care can achieve.

Postoperative rehabilitation

The impact of robotics resonates beyond the confines of the operating room, extending to the rehabilitation phase. Robotics-assisted rehabilitation devices, including exoskeletons and motion-capture systems, are revolutionizing how patients regain mobility and strength after orthopedic procedures [6]. These devices provide targeted exercises, monitor progress, and adjust the rehabilitation regimen based on real-time data, thus ensuring optimal recovery trajectories. Patients partake in a more engaged and effective rehabilitation journey, yielding enhanced long-term outcomes.

Challenges and ethical considerations

While integrating robotics into orthopedic healthcare holds great promise, it concurrently presents challenges and ethical considerations. The initial expenses associated with procuring and implementing robotic systems can be substantial, engendering concerns about accessibility and healthcare inequities. Furthermore, specialized training to proficiently operate these systems necessitates ongoing education and skill development investments. Ethical dilemmas arise in cases where the equilibrium between human expertise and robotic assistance is at issue. Striking the right balance between surgeon intervention and robotic autonomy necessitates meticulous deliberation. The focus should invariably remain on safeguarding patients’ well-being and ensuring that the benefits of automated technologies outweigh any potential risks.

The path ahead

As the realm of robotics in orthopedic healthcare continues its expansion, a proactive and visionary approach is indispensable. Collaborative endeavors involving engineers, medical practitioners, and regulatory authorities are pivotal in refining existing technologies and conceiving novel solutions that cater to the evolving needs of patients. Investments in research and development will be instrumental in fostering innovation, thereby propelling the creation of even more sophisticated robotic systems capable of redefining the boundaries of orthopedic care.

Integrating robotics into orthopedic healthcare marks a watershed moment in the medical arena. Its precision, personalization, and minimally invasive approach reshape the landscape of orthopedic procedures, offering patients renewed hope and an enhanced quality of life. As the medical community, policymakers, and technology innovators navigate the challenges and ethical considerations, the collective responsibility lies in shaping a future where robotics and human expertise harmoniously converge, redefining the horizons of orthopedic healthcare. With each surgical procedure and rehabilitation session, robotics propels us closer to a world where musculoskeletal conditions are not merely treated but unequivocally conquered.
